# Potential Mechanism for HIV-Associated Depression: Upregulation of Serotonin Transporters in SIV-Infected Macaques Detected by 11C-DASB PET

**DOI:** 10.3389/fpsyt.2019.00362

**Published:** 2019-05-23

**Authors:** Swati Shah, Sanhita Sinharay, Kenta Matsuda, William Schreiber-Stainthorp, Siva Muthusamy, Dianne Lee, Paul Wakim, Vanessa Hirsch, Avindra Nath, Michele Di Mascio, Dima A. Hammoud

**Affiliations:** ^1^Center for Infectious Disease Imaging (CIDI), Radiology and Imaging Sciences,Clinical Center, National Institutes of Health (NIH), Bethesda, MD, United States; ^2^Laboratory of Molecular Microbiology, National Institute of Allergy and Infectious Diseases (NIAID), NIH, Bethesda, MD, United States; ^3^Biostatistics and Clinical Epidemiology Service, Clinical Center, NIH, Bethesda, MD, United States; ^4^National Institute of Neurological Disorder and Stroke (NINDS), NIH, Bethesda, MD, United States; ^5^AIDS Imaging Research Section, Division of Clinical Research, NIAID, NIH, Rockville, MD, United States

**Keywords:** HIV, SIV, depression, serotonin transporter, PET

## Abstract

**Purpose:** Increased incidence of depression in HIV+ patients is associated with lower adherence to treatment and increased morbidity/mortality. One possible underlying pathophysiology is serotonergic dysfunction. In this study, we used an animal model of HIV, the SIV-infected macaque, to longitudinally image serotonin transporter (SERT) expression before and after inoculation, using 11C-DASB (SERT ligand) PET imaging.

**Methods:** We infected seven rhesus macaques with a neurovirulent SIV strain and imaged them at baseline and multiple time points after inoculation (group A). Pyrosequencing methylation analysis of the SERT promoter region was performed. We also measured SERT mRNA/protein in brain single-cell suspensions from another group (group B) of SIV-infected animals (n = 13).

**Results:** Despite some animals showing early fluctuations, 86% of our group A animals eventually showed a net increase in midbrain/thalamus binding potential (BP_ND_) over the course of their disease (mean increased binding between last time point and baseline = 30.2% and 32.2%, respectively). Repeated-measures mixed-model analysis showed infection duration to be predictive of midbrain BP_ND_ (p = 0.039). Thalamic BP_ND_ was statistically significantly associated with multiple CSF cytokines (P < 0.05). There was higher SERT protein levels in the second group (group B) of SIV-infected animals with SIV encephalitis (SIVE) compared to those without SIVE (p = 0.014). There were no longitudinal changes in SERT gene promoter region percentage methylation between baselines and last time points in group A animals.

**Conclusion:** Upregulated SERT leading to lower synaptic levels of serotonin is a possible mechanism of depression in HIV+ patients, and extrapolating our conclusions from SIV to HIV should be sought using translational human studies.

## Introduction

Despite mounting evidence of higher depression rates in HIV-positive (HIV+) individuals compared to seronegative controls and associated increased morbidity and mortality (1), there is limited literature targeting the underlying mechanisms of depressive disorders in HIV. In one paper, the levels of serotonin transporter (SERT) mRNA in the peripheral blood mononuclear cells (PBMCs) of SHIV-infected rhesus macaques were significantly reduced compared to control animals, suggesting that SERT expression might be affected in HIV (2). In another study, disruption of cytoskeletal genes and dysregulation of somatostatin were found to be part of the pathologic process of major depressive disorder (MDD) in the setting of HIV (3). Tryptophan metabolism dysregulation is also suspected to play a role (4, 5).

The only previous positron emission tomography (PET) study targeting SERT in the setting of HIV depression demonstrated generally lower 11C-DASB (radioactive ligand targeting SERT) binding in HIV+ patients compared to healthy controls. Depressed HIV+ patients, however, showed higher 11C-DASB binding than non-depressed patients, suggesting SERT upregulation in the depressed group and possible abnormal serotonergic transmission in HIV-associated depression (6). No other similar cross-sectional or longitudinal PET studies have been performed to date.

In the current study, we used PET and 11C-DASB to assess SERT binding in an HIV animal model, the SIV-infected macaque, at baseline and at different intervals after inoculation with a neurotropic SIV strain (SIVsm804E) to determine the natural history of serotonergic dysregulation in relation to serum and CSF markers of SIV infection. We correlated our findings with clinical and laboratory markers of SIV infection and performed a detailed analysis of the SERT gene promoter region methylation changes between baseline and the last time point to assess any potential epigenetic effects on gene expression. Based on previous literature describing *in vitro* and *in vivo* interactions between various cytokines (e.g., IL-1, IL-6, IL-10, TNFα, and IFNγ) and SERT expression (7–12), we specifically correlated 11C-DASB binding with levels of various CSF cytokines.

## Methods and Materials

### Subjects

All procedures were performed in accordance with the recommendations of the *Guide for the Care and Use of Laboratory Animals*. The study was approved by the Animal Care and Use Committee of the National Institutes of Allergy and Infectious Diseases (NIAID), National Institutes of Health (NIH).

Fifty Indian rhesus macaques (*Macaca mulatta*) were genotyped for Trim5α. Seven animals were found to have the Q/Q genotype (group A, **Supplementary Table 1S**) and thus were selected for this study, as they are known to have increased susceptibility to development of neurological disease (13–15). All animals were inoculated intravenously with 500 TCID_50_ (50% tissue culture infective doses) of SIVsm804E (14). The selected animals included five females and two males (mean baseline age = 3.7 years). Five out of seven animals (SIV #1, 2, 3, 4, and 7) progressed soon after inoculation (mean = 14.6 weeks, “rapid progressors”) and were rescued with treatment (daily subcutaneous injections of tenofovir (PMPA; 20 mg/kg) and emtricitabine (FTC; 30 mg/kg) with raltegravir (20 mg/kg) mixed with food twice per day). Two of the rapid progressors succumbed to the disease despite treatment (SIV #4 and 7), while the others responded to treatment and survived. The two “slow progressors” (SIV #5 and 6) did not show symptoms until 87 and 91 weeks after inoculation, respectively. On development of symptoms, one slow progressor (SIV #6) responded to treatment, while the other subject (SIV #5) developed a presumed opportunistic infection and had to be excluded.

Treatment was eventually discontinued, and the surviving animals (one slow and three rapid progressors) were allowed to progress to a chronic infectious stage prior to necropsy. The last imaging time point, however, did not always correspond to the survival of the animals, due to logistical considerations. Details of group A animals’ imaging and disease progression are included in **Table 1S**.

We also evaluated whole-brain cell suspensions obtained from a separate group of SIV-infected monkeys (group B, n = 13), of which only six animals showed neurological symptoms and were found to have neuropathology consistent with SIVE, as described earlier (16).

### Magnetic Resonance Imaging

All subjects underwent magnetic resonance (MR) imaging using a 3T Achieva Philips scanner (Philips Healthcare, Best, Netherlands) and a three-dimensional (3-D) MPRAGE sequence with the following parameters: repetition time = 7.77 ms, echo time = 3.45 ms, echo train length = 128, flip angle = 9°, number of excitations = 1, field of view = 12 × 12 cm, matrix = 256 × 256, and slice thickness = 0.5 mm. Baseline and follow-up MR scanning was performed within a few days of every PET session to rule out structural abnormalities that could affect the PET results.

For both the MRI and PET studies, the animals were anesthetized using a combination of ketamine (∼0.1 ml/kg) and propofol (0.2 mg/kg/min). The concentrations of various anesthetics as well as the timing of administration with respect to imaging were kept constant across the studies. Over the entire imaging session, the animals were carefully monitored for changes in body temperature, spO_2_, and heart and respiratory rates.

### PET Imaging

PET images were acquired on a CPS/CTI High Resolution Research Tomograph (HRRT), head-only camera (17). Prior to radiotracer injection, a 6-min transmission scan was obtained for calculation of attenuation correction maps. 11C-DASB was synthesized as previously described (18). Following intravenous bolus administration of 11C-DASB (mean injected dose = 5.67 ± 0.61 mCi), a dynamic 120-min-long emission scan was acquired using a 50-frame protocol. PET scans were reconstructed using the ordered subsets expectation maximization (OSEM) algorithm, in a 31 × 31 cm field of view and 256 × 256 pixel matrix with pixel size of 1.2 × 1.2 mm. PET frames were corrected for radioactive decay. The image frames were then co-registered to each animal’s structural MRI image using Pmod 3.7 software (PMOD Technologies LLC, Zurich, Switzerland). There was a minimum gap of 5 weeks between consecutive PET scans in the same animals (range of 5–38 weeks).

### PET Data Analysis

The reconstructed PET images were first co-registered to their respective MR images using rigid body transformation. Volumes of interest (VOIs) were selected based on a monkey brain template overlaid on the MR images and readjusted manually as necessary. One set of VOIs (for the midbrain, thalamus, caudate, and putamen) adjusted for the baseline study was reapplied on follow-up MRI scans to maintain consistency in measurements. One VOI was also selected in the cerebellar cortex, avoiding the cerebellar white matter and cerebellar vermis, to be used as a reference region (19). The outcome measure in our study was SERT binding potential normalized to non-displaceable tissue radioligand (BP_ND_), measured using a simplified reference tissue model (SRTM) (20) with the cerebellar cortex as a reference region (19). Although VOIs were drawn in the midbrain, thalamus, caudate, and putamen, only thalamic and midbrain values were used for statistical analysis due to their relevance to disease pathophysiology. Mean voxel BP_ND_ values for each VOI were extracted from the scans and compared over time. Parametric maps were generated using the Pixel-wise modeling tool (PXMOD) in PMOD.

We performed a total of 27 PET scans on the 7 macaques, including 7 baseline and a total of 20 follow-up scans (**Table 1S**).

### Specimen Collection

#### Cytokine/Chemokine Level Measurements in the CSF

Cytokine/chemokine levels in the CSF were obtained to assess the potential relationship between CSF neuroinflammatory markers and SERT expression levels as assessed by PET imaging. Concentrations of MCP-1, TNFα, IFNγ, IL-1ra, IL-2, IL-6, IL-8, IL-10, IL-18, GCSF, sCD40L, and VEGF were measured in the CSF of five infected animals within a few days of each PET scan, using a bead-based multiplex assay (EMD Millipore). The assay was performed according to the manufacturer’s instructions. The assay plates were read on the Bio-plex 200 System (Bio-Rad).

#### PBMC Collection

We collected blood specimens from our animals (group A) corresponding to their imaging sessions in order to assess viral load, cell counts (flow cytometry), and peripheral SERT mRNA and to evaluate for potential peripheral epigenetic changes corresponding to changes in SERT expression. Blood collected was used for PBMC isolation using Ficoll. The PBMCs were re-suspended in cell freezing medium at 10 million cells/vial and stored in liquid nitrogen until needed. DNA/RNA was isolated from the cells using the ZR-Duet DNA/RNA Miniprep kit (Zymo Research) as per the manufacturer’s instructions for downstream applications.

#### Preparation of Whole-Brain Cell Suspensions

Due to our small sample number of imaged animals and since the brain tissues of those animals could not be used for detailed PCR and Western analysis of SERT expression, we decided to evaluate another group of animals that were similarly infected and assessed for development of SIV encephalitis in another study (16). Whole-brain single-cell suspensions had already been obtained from a separate group of SIV-infected monkeys (group B, n = 13). Following saline perfusion of the animals, fresh brain tissues were collected from the frontal, parietal, and temporal lobes, the cerebellum, and the midbrain during necropsy. Multiple pieces of brain tissue from each region were then homogenized and pooled to obtain a cell suspension representative of the whole brain (14). RNA was extracted from these samples using the RNeasy Lipid Tissue Mini Kit from Qiagen. Protein lysates were obtained by re-suspending the cells in cold RIPA buffer with protease inhibitors (Roche). The samples were then vortexed and centrifuged at 14,000 rpm for 30 min at 4°C to remove debris.

### Specimen Analysis

#### Flow Cytometry

Absolute blood CD3, CD4, and CD8 T-cell counts as well as % Ki67 in CD4 and CD8 T-cells were measured as previously described (14).

#### qPCR

qPCR was performed to assess SERT mRNA levels in PBMCs derived from group A animals, in order to evaluate for potential peripheral changes of SERT transcription after inoculation. qPCR was also performed to assess SERT mRNA levels in whole-brain single-cell suspensions from group B animals, in order to correlate with the incidence of SIV encephalitis. cDNA was synthesized using the RT^2^ First Strand (Qiagen). The qPCR assay was set up using RT² SYBR Green qPCR Mastermix (Qiagen) to measure SERT (Qiagen) and normalized to *rpl13a* (Qiagen). The plates were run on the CFX96 real-time qPCR system (Bio-Rad). For data analysis, we calculated fold change using the comparative C_T_ method as previously described (21).

#### Western Blotting

Western blotting was performed to assess SERT protein levels in whole-brain single-cell suspensions from group B animals. Protein lysates from whole-brain cell suspensions (group B) were used for Western blotting as previously described (22). The primary antibodies used were SERT (Abcam) at 1:300 and GAPDH (CST) at 1:1,000 dilutions. The secondary antibody, goat anti-rabbit (Jackson), was used at 1:50,000. ImageJ was used to quantify the band intensities from scanned blots. Results are shown as fold change with respect to the average of animals with no SIVE.

### Epigenetic (Methylation) Analysis

Pyrosequencing analysis was performed for the promoter region of the *SLC6A4* gene by Qiagen (GmbH Hilden, Germany) on DNA from group A animals, sampled at baseline and multiple time points between inoculation and euthanasia. Briefly, DNA extracted from the PBMCs of macaques (n = 7) was used for bisulfite conversion using the Epitect Fast Bisulfite Conversion Kit (Qiagen). The promoter region of *SLC6A4* was amplified from 20 ng of treated DNA using the primers (TAGAGTTAGGAGGGGAGGGAT) and (ACACCAACAAACCCCTAT). This was followed by sequencing using the primer (AGGAGGGGAGGGATT) with PyroMarkQ24 Advanced (Qiagen). A total of nine CpG islands were analyzed in the promoter region.

To assess global methylation changes, a Methylated CpG Island Recovery Assay (MIRA) was used as previously described (23). The assay enriches methylated CpG islands based on high-affinity interactions to methyl-binding protein complexes using the MethylCollector Ultra Kit (ActiveMotif). PBMC DNA of group A (n = 7) macaques (baseline and last time points) were used and sequenced using the Illumina Platform (NIH Intramural Sequencing Center). Data analysis was done by Acura Science (Iowa, USA). FASTQC was used for quality control of the sequencing data. Reads were aligned to the rhesus reference genome (Ensembl release 88). Methylation peaks were subsequently identified using MACS2. Differential methylation analysis was performed as before (24). Peak “summits”—i.e., the single points representing the center point of the peaks—located within 600 bp were grouped together and then merged (extended peaks) using a locally developed Perl script. Normalized counts for each extended peak were calculated as total count in this region divided by the length of the extended peak and then by the number of reads mapped in the sample. Differentially methylated regions (DMRs) were defined as extended peaks with large fold changes (>1.5) in normalized counts, and only DMRs covered by five or more reads in the samples being compared were considered. Each DMR was annotated into the following categories: exon, intron, transcription start site (TSS), promoter (−1 kb and +100 bp of a gene’s TSS), 5’ UTR, 3’ UTR, intergenic, and non-coding.

### Statistical Analysis

Statistical analysis was performed using SAS, version 9.4 (SAS Institute, NC, USA), and Prism (GraphPad, version 7.01).

Longitudinal changes of midbrain and thalamus BP_ND_ values were plotted for every animal, and the percentages of differential binding between the last time point and baseline were calculated. Repeated-measures mixed models were used to predict 11C-DASB BP_ND_ in the midbrain and thalamus of group A animals, based on CSF VL, plasma VL, the duration of infection, CD4, CD3, CD8 T-cell counts, and % Ki67 in CD4 and CD8 T-cells. CSF concentrations of MCP-1, IL-1ra, IL-2, IL-6, IL-10, IL-18, GCSF, sCD40L, and VEGF were available for five animals and were included as potential predictors. Each measurement was first included individually in a statistical model as an explanatory variable, with BP_ND_ as the response variable. We then included all measurements in one model to assess combined relationship with BP_ND_. A mixed model was fit to the data because of the repeated-measures nature of the data. Several variance–covariance matrix structures were considered for each model, and the one with lowest Bayesian information criterion (BIC) was applied. Model-fit diagnostics were examined to check whether model assumptions were met. Because this is an exploratory study with a relatively small sample size, no multiple-comparisons adjustment was used (all reported p-values are unadjusted).

For group B animals, we compared the fold increase in SERT protein and mRNA of SIVE (n = 6) vs. non-SIVE animals (n = 7) using the Mann–Whitney test.

All data are represented as mean ± SD except for group B qPCR and Western results, which are displayed as median values ± interquartile range (IQR).

## Results

### PET Imaging Analysis

Mean BP_ND_ values in the selected VOIs were highest in the midbrain, followed by the thalamus, caudate/putamen, and cerebellum, which is similar to reported postmortem SERT densities (25). Even though SIV involves the whole brain, including the cerebellum, the lack of specific uptake in the cerebellar cortex still warrants its use as a reference region (20, 26).

**Figure 1:**
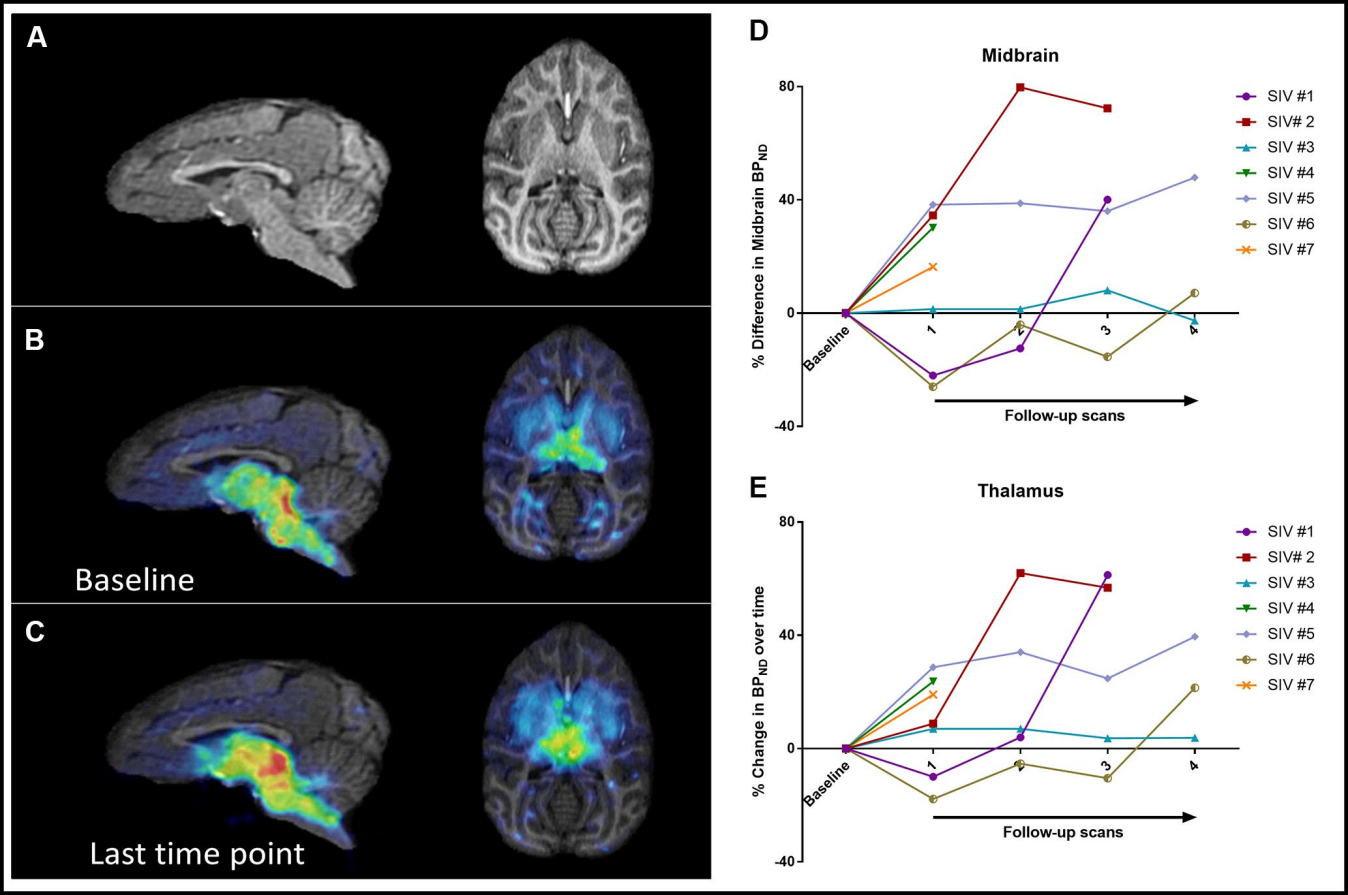
Sagittal and axial brain MRI images **(A)** as well as parametric maps of 11C-DASB BP_ND_ at baseline **(B)** and last time point **(C)** of SIV #4. Increased BP_ND_ can be detected visually at the last time point compared to baseline. Changes in BP_ND_ over the course of disease in all seven animals in the midbrain **(D)** and thalamus **(E)**.

We concentrated on two high-binding regions, the midbrain and thalamus, due to their relevance to disease pathophysiology. Regional midbrain BP_ND_ values at 5–10 weeks after inoculation increased in four animals (16–38%) and decreased in two animals (22–26%). One animal did not show substantial change. Over the course of disease, six out of seven subjects showed a net increase in midbrain BP_ND_ values at the last time point compared to baseline (range: 7.1–72.4%), while one subject showed a minimal decrease (−2.6%) (**Figure 1**). The average change in all seven animals was an increase of 30.2% ± 25.8% over the course of disease.

Thalamic BP_ND_ values at 5–10 weeks after inoculation increased in five animals and decreased in two animals. Over the course of disease, all animals showed a net increase in BP_ND_ at the last time point (32.2% ± 21.1%), although in one animal, the increase was minimal (3.8%) (**Figure 1**).

**Figure 2:**
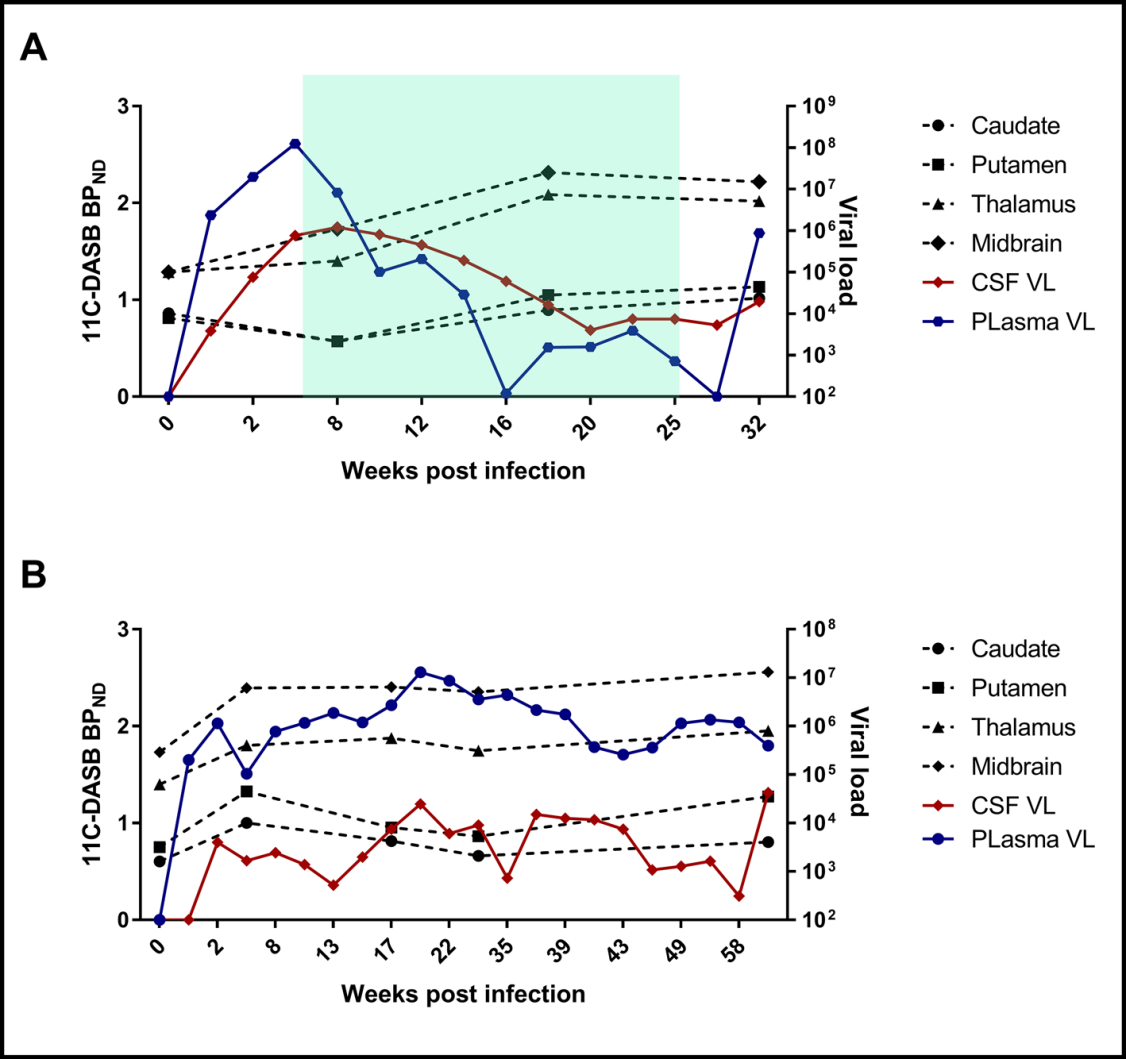
Examples of changes of BP_ND_ values at different time points with respect to CSF and plasma VL changes in one fast progressor, SIV #2 **(A)**, and one slow progressor, SIV #5 **(B)**. The green shadow in **(A)** reflects the duration of antiretroviral treatment.

A repeated-measures mixed-model analysis taking into account all the measured time points showed that among the selected variables, duration of infection correlated positively with 11C-DASB midbrain BP_ND_ (p = 0.039), with a positive trend also observed between duration of infection and thalamic BP_ND_ (p = 0.081). Neither treatment initiation nor interruption had a consistent effect on BP_ND_ (**Figure 2**). There were no significant correlations between BP_ND_ and CD4, CD3, CD8 T-cell counts, or % Ki67 in CD4 or % Ki67 in CD8 T-cell counts.

### Cytokine/Chemokine Measurements

CSF cytokine analysis showed an increase in MCP-1, TNFα, IFNγ, IL-1Ra, IL-2, IL-6, IL-8, IL-10, Il-12, and IL-18 concentrations after inoculation in all animals, which corresponded to a concomitant increase in CSF VL. In four treated animals, cytokine levels decreased after treatment and rebounded after interruption of treatment, as expected.

There were significant positive correlations between CSF levels of MCP-1, IL-1Ra, IL-6, IL-8, IL-10, and IL-18 and thalamic BP_ND_ (p = 0.002, 0.003, 0.035, 0.013, 0.023, and 0.014, respectively). No significant correlations between CSF cytokines and midbrain BP_ND_ were found.

### Expression of SERT in the Periphery and the Brain

#### Group A Animals

**Figure 3:**
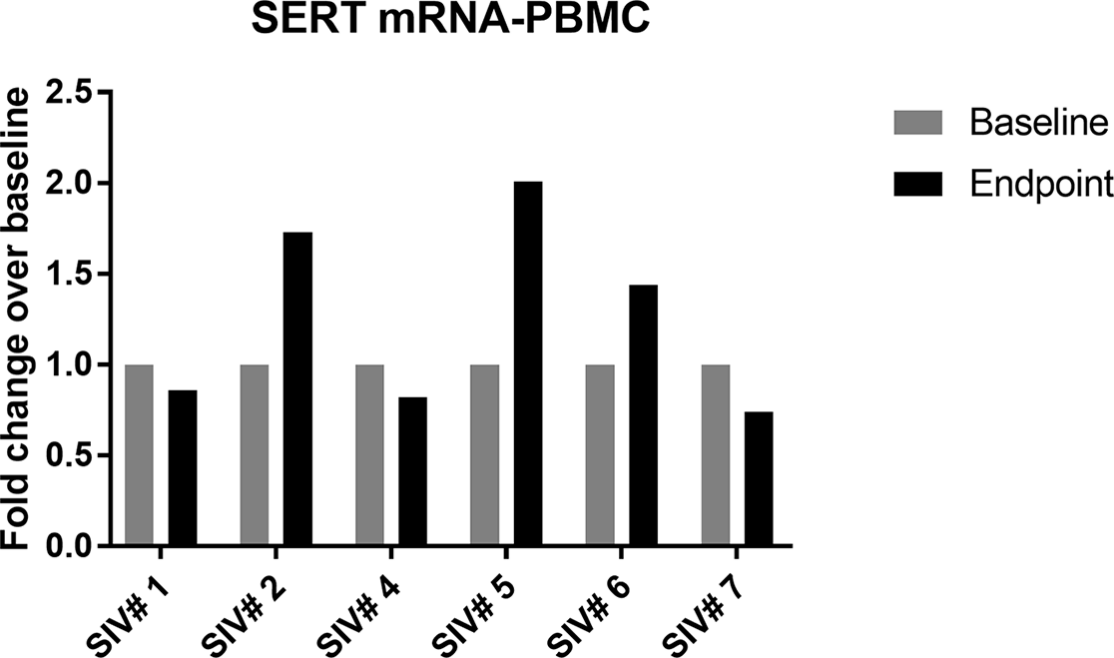
Change in SERT mRNA levels in PBMCs of infected animals (group A) between baseline and last time point; 50% of the animals showed slight increase in SERT mRNA compared to baseline values.

The expression of SERT mRNA in PBMCs was very low across all samples and undetectable in one animal (SIV #3). It increased slightly in three out of six animals at the last time point compared to baseline (**Figure 3B**).

#### Group B Animals

**Figure 4:**
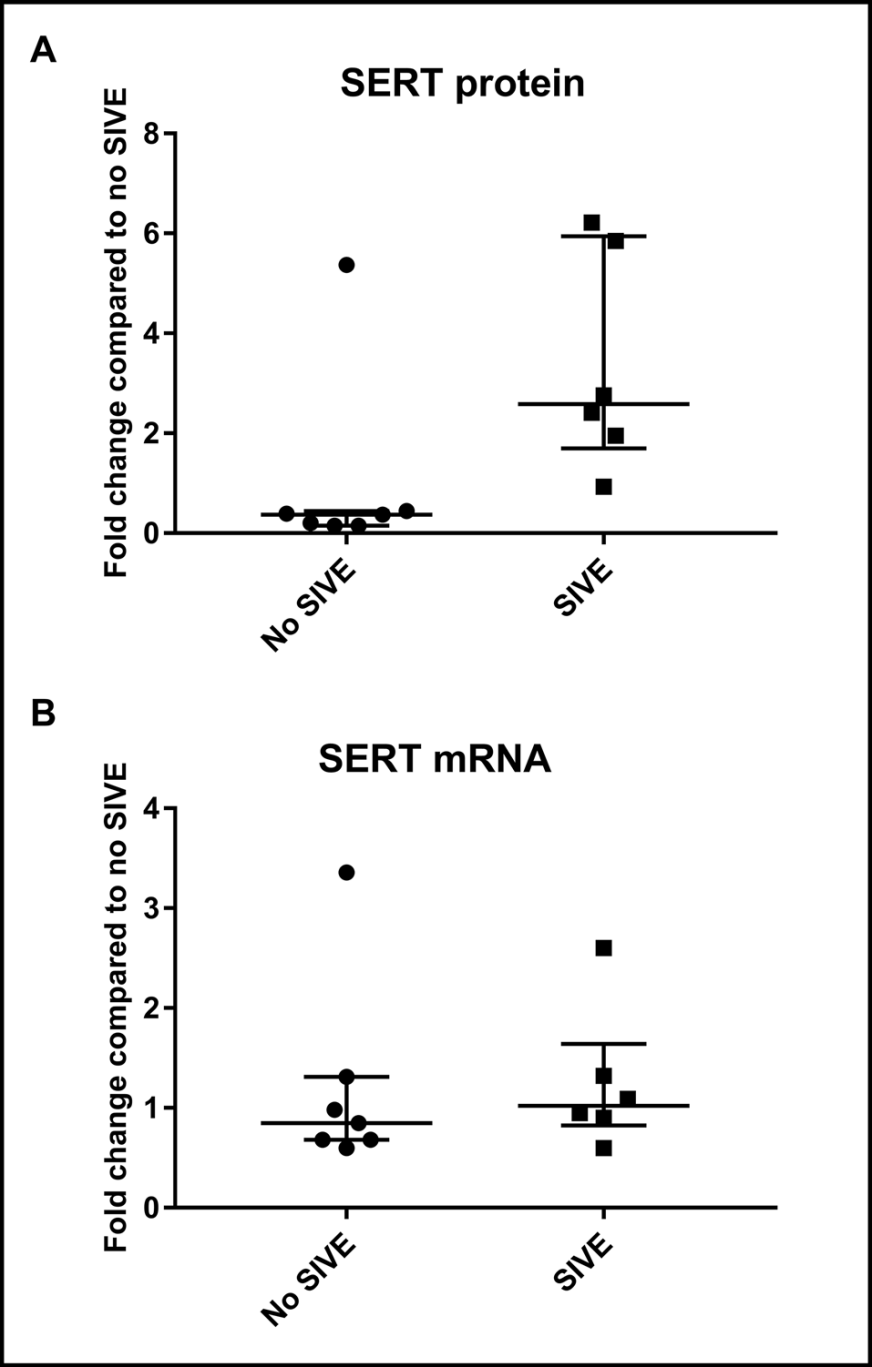
Western blotting and qPCR results in group B animals (whole-brain lysates). **(A)** Fold change in protein levels of SIVE compared to non-SIVE animals. **(B)** Fold change in mRNA expression of SIVE compared to non-SIVE animals. Median and interquartile range values are shown (* = p < 0.05; n.s. = non-significant).

SERT protein levels in whole-brain lysates of group B animals were compared between infected animals that showed neurological symptoms associated with the development of SIVE (n = 6) and a group that was infected but did not display neurological dysfunction (n = 7). The values were normalized to the average of animals with no SIVE. Expression of mRNA normalized to a housekeeping gene (*rpl13A*) was also compared between SIVE (n = 6) and non-SIVE animals (n = 7), with data represented as fold increase with respect to the average of non-SIVE animals. There was no significant change in SERT mRNA expression (average increase = 1.24-fold, p = 0.55); however, there was a significant increase in SERT protein expression (average increase = 3.35-fold, p = 0.014) (**Figure 4**).

### Epigenetic (Methylation) Analysis

To assess whether the observed increases in SERT expression were mediated by epigenetic regulation, our pyrosequencing analysis was focused on evaluating the percent methylation of 9 CpG islands closest to the TSS of the *SLC6A4* (SERT) gene, isolated from PBMC DNA of seven macaques taken at various time intervals starting with baseline and continuing until the terminal time point. In each case, the percent methylation was less than 5%, indicating there was hardly any methylation in the promoter region of SERT (**Supplementary Table 1S**). We did not find any appreciable change in percent methylation between baseline and multiple time points after inoculation.

The MIRA-seq analysis of the whole genome was done to identify potential epigenetic modifications to other upstream factors that could affect SERT expression and trafficking. Methylated CpG islands across the entire genome were assessed for differentially methylated regions (DMR). An average of 91.4% of the reads were mapped back to the genome. Based on the differential methylation analysis obtained for the baseline and the last time point, fold change was calculated, and only genes that met the cutoff of >1.5 (hypermethylated) and <0.5 (hypomethylated) were considered. In five out of the seven animals, the promoter region (−350 to −390 from TSS) of the transcription factor deformed epidermal autoregulatory factor-1 (*DEAF1*) was differentially methylated. *DEAF1* was hypermethylated in three animals at the last time point compared to baseline, with a fold change (FC) range of 1.6–2.2. On the other hand, *DEAF1* was slightly hypomethylated in one animal that succumbed to the disease very early on (FC = 0.4) and another animal that survived and was treated (FC = 0.7). There were no consistent methylation peaks captured for two animals in the *DEAF*1 promoter region, though we saw some peaks in the intragenic regions at the terminal time point.

## Discussion

Using 11C-DASB high-resolution PET imaging, we have documented longitudinal *in vivo* increases in SERT expression in 85% of SIV-infected macaques when we compared baseline BP_ND_ levels to multiple time points between inoculation and euthanasia. In a mixed-effect analysis model, midbrain BP_ND_ values for 11C-DASB correlated significantly with duration of infection, although we did not find significant correlations to CSF/plasma VL or various T-cell counts. There were also significant positive correlations between thalamic BP_ND_ and CSF levels of MCP-1, IL-6, IL-8, and IL-18, cytokines with pro-inflammatory properties, as well as IL-1Ra and IL-10, both anti-inflammatory cytokines. Our imaging data were further supported by findings of increased SERT protein levels in another set of SIV-infected animals with symptoms of SIVE compared to asymptomatic infected animals.

Higher frequency of depression has been repeatedly documented in HIV+ compared to HIV-negative (HIV-) subjects: a 2001 meta-analysis found the frequency of MDD to be nearly two times higher in HIV+ subjects (27), with more recent work confirming that figure (1, 28, 29). Beyond the psychological toll of depression in HIV+ subjects, the ramifications extend to its impact on survival, mainly through its effect on treatment adherence (30) and secondary control of the infection: untreated depression was associated with significantly decreased odds of achieving >90% adherence to HAART and significantly lower odds of controlling HIV RNA levels to <500 copies/ml (31). In another study, somatic symptoms of depression were associated with shortened survival in HIV+ patients (32). More recently, depression was found to be associated with increased risk of missing appointments, increased risk of a detectable viral load, and a doubled mortality rate (1). Despite the magnitude of the problem, the mechanisms underlying higher depression rates in HIV remain poorly understood. Previous studies suggested disturbances of tryptophan metabolism and SERT expression both *in vitro* and *in vivo* (2, 4–6). The only previous imaging study assessing the serotonergic system in HIV showed higher 11C-DASB BP_ND_ in depressed compared to non-depressed HIV+ patients (6).

Upregulation of SERT reflected by increased 11C-DASB BP_ND_ values has been described in subjects with MDD and bipolar disease (33), Parkinson’s patients with depression (34), and patients with highly negativistic dysfunctional attitudes (35), among others. A possible explanation for depressive symptomatology in the setting of upregulated SERT is exaggerated serotonin reuptake into the presynaptic neurons, which subsequently leads to decreased serotonin levels in the synapse.

Our current results are not consistent with previous work where we found generally decreased 11C-DASB binding in HIV+ patients compared to controls (6). Those subjects, however, had been infected with HIV for a much longer period of time than our animals, which could have resulted in neuronal loss. Interestingly, the depressed HIV+ patients in that study showed higher 11C-DASB binding than non-depressed subjects, pointing towards a connection between SERT expression and depressive symptomatology.

What could be the cause of increased SERT expression in SIV/HIV? There is a large body of literature describing interactions between various cytokines (e.g., IL-1, IL-6, IL-10, TNFα, and IFNγ) and SERT expression, both *in vitro* and *in vivo* (7–12). For example, TNFα was found to enhance the transport capacity of SERT-specific serotonin uptake in primary astrocytes, consistent with prior observation of an increase in SERT mRNA levels (10). This seemed to be mediated through activation of the p38 mitogen-activated protein kinase (MAPK) signaling pathway, since pre-treatment with a p38 MAPK inhibitor attenuated the TNFα-mediated stimulation of serotonin transport (10). In support of this potential interaction, we have found significant positive correlations between thalamic BP_ND_ and CSF levels of multiple pro-inflammatory and anti-inflammatory cytokines. Interestingly, the cytokine correlations with the midbrain BP_ND_ were not significant. The reason for this discrepancy is unclear, although similar findings have been previously reported by another group where only thalamic and not midbrain SERT availability was correlated with IL-10 in bipolar disorder (11). One possible explanation is that thalamic neurons are in closer contact with the CSF and thus could be more affected by CSF cytokines (36).

Another connection between HIV and SERT upregulation could be through the production of SIV/HIV viral proteins. In mice, a single exposure to Tat in the brain was enough to induce brain cytokine signaling that resulted in depressive-like behavior (37). In a paper by Fu et al., Tat increased the expression of SERT in organotypic hippocampal slice cultures, an effect that was attenuated by pre-treatment with SB 202190, a p38 MAPK inhibitor (38). Prolonged exposure to viral proteins in our animals could therefore have contributed to increased SERT expression over time.

We hypothesized that the changes we observed in our SIV-infected monkeys could also be a result of epigenetic modulation. Epigenetic modifications induced by HIV have been previously described, especially related to accelerated aging and viral latency (39–41). On the other hand, depression in general and changes in SERT expression specifically have been found to be associated with epigenetic modifications, especially changes in DNA methylation (42–45). In our evaluation of SERT gene methylation, however, we did not find significant changes in methylation of SERT promoter between the pre-infected and latest time point in our imaged animals. Looking for potential interactive epigenetic changes upstream from SERT throughout the whole genome, we found one gene, *DEAF1*, which seemed to be differentially methylated in five out of the seven animals. DEAF1 regulates the transcription of multiple genes and has been implicated in type 1 diabetes (46), cancer (47), and IFNβ production (48). It is interesting to note that, since increased IFNβ secretion can restrict HIV viral replication and spreading (49, 50), the silencing of *DEAF1* can confer a significant advantage in maintaining the potency of infection. Additionally, a separate function of DEAF1 is to act as a repressor of serotonin receptor subtype 1A (5-HT1A) in non-neuronal and presynaptic raphe cells (51–53) and an enhancer of 5-HT1A in other neuronal cells (54). Therefore, hypermethylation of *DEAF1* could potentially reduce its availability to repress 5-HT1A autoreceptor expression, which in turn is linked to depression (55, 56). Multiple reports have shown that dysregulation of 5-HT1A function can have a secondary effect on SERT expression: Bose et al. showed that presynaptic 5-HT1A expression is related to SERT density in specific regions of the brain (57), while another study used autoradiography to demonstrate a concomitant decrease of SERT expression in the basal ganglia, thalamus, and cortical regions of 5-HT1A knockout mice (58). SIV-induced epigenetic alterations to DEAF1 expression could thus possibly play a role in the perceived changes in SERT expression. However, our current findings do not support a definite role, and further work is needed to better elucidate the interplay between DEAF1 signaling and SERT expression. Additionally, our results do not preclude the effect of other epigenetic and non-epigenetic mechanisms on SERT expression.

Our study is limited by a relatively small size number of SIV-infected monkeys. As such, our results should be interpreted with caution, as this is an exploratory study. We also did not have fresh brain tissue from the imaged animals to systematically assess SERT mRNA and protein levels in correlation with 11C-DASB binding. Most importantly, we did not collect neuropsychological or behavioral measures of depression in the animals, mainly because there is no clear consensus on what constitutes depressive-like behavior in animals compared to humans and because in our animals, the presence of a severe infectious process confounds the detection of subtle psychiatric manifestations. Even though we did not have control animals followed over the same period of time, we have used longitudinal imaging with each animal acting as its own control. We do not believe that scanning-related stress could have affected our results, since the scans were separated by a minimum gap of 5 weeks (range: 5–38 weeks). Finally, we have not looked for methylation changes in the brains of the infected animals, but rather, in the periphery, which allowed us to perform a longitudinal assessment. It has been previously shown, however, that there is a significant correlation between the methylation levels in the brain and peripheral blood (59).

In conclusion, we have identified increased expression of SERT in six out of seven SIV-infected monkeys using *in vivo* PET imaging, which correlated with duration of infection in the midbrain. The change in expression in the thalamus also correlated with pro- and anti-inflammatory cytokine activity in the CSF. Although we did not find direct methylation changes involving the SERT promoter gene to explain our results, scouring of the whole-genome methylation status resulted in a potential connection between a differentially methylated gene, *DEAF1*, and upregulated SERT expression. Whether this connection will prove to be widespread is unclear, and the possibility of other epigenetic factors affecting SERT expression in SIV cannot be ruled out. Extending our conclusions from SIV to HIV needs further confirmation with translational HIV human studies. More work is also needed to better understand the connection between SERT upregulation and depression symptomatology.

## Ethics Statement

All procedures were performed in accordance with the recommendations of the Guide for the Care and Use of Laboratory Animals. The study was approved by the Animal Care and Use Committee of the National Institute of Allergy and Infectious Diseases (NIAID), National Institutes of Health (NIH).

## Author Contributions

DH, KM, DL, VH, AN, and MDM conceived of and designed the study. DH, SSh, SSi, WS-S, KM, SM, and DL evaluated the study subjects and/or collected and analyzed the data. DH and PW performed the statistical analysis. All authors participated in drafting the article and/or revising it critically for intellectual content and gave final approval of the submitted manuscript.

## Funding

Funding for this study was provided in part by the intramural research program of the National Institute of Allergy and Infectious Diseases (NIAID), National Institutes of Health (NIH), and by the Center for Infectious Disease Imaging (CIDI), Clinical Center, NIH. This project has also been funded in part with federal funds from the National Cancer Institute, NIH, under contract no. HHSN261200800001E. The content of this publication does not necessarily reflect the views or policies of the Department of Health and Human Services, nor does the mentioning of trade names, commercial products, or organizations imply endorsement by the U.S. government.

## Conflict of Interest Statement

The authors declare that the research was conducted in the absence of any commercial or financial relationships that could be construed as a potential conflict of interest.

The reviewer CL declared a shared affiliation, with no collaboration, with one of the authors AN to the handling editor.

## Supplementary Material

Table 1S Characteristics of group A animals.

Table 2S Characteristics of group B animals.

Table 3S Percent methylation of 9 CpG islands closest to the transcription start site (TSS) of the *SLC6A4* (SERT) gene from PBMC DNA of 7 macaques collected at various time intervals starting with baseline (before inoculation) up to the terminal point.

## References

[1] Pence BW Mills JC Bengtson AM Gaynes BN Breger TL Cook RL Association of increased chronicity of depression with HIV appointment attendance, treatment failure, and mortality among HIV-infected adults in the United States. JAMA Psychiatry (2018) 75(4):379–85. 10.1001/jamapsychiatry.2017.4726 PMC5875308 29466531

[2] Yu K Qiu CL Yang GB Zong CM Xing H Shao Y Alteration of serotonin transporter messenger RNA level in the peripheral blood mononuclear cells from simian/human immunodeficiency virus infected Chinese rhesus macaques (Macaca mulatta). Brain Behav Immun (2010) 24:298–305. 10.1016/j.bbi.2009.10.008 19854262

[3] Everall IP Salaria S Atkinson JH Young C Corbeil J Grant I Diminished somatostatin gene expression in individuals with HIV and major depressive disorder. Neurology (2006) 67:1867–9. 10.1212/01.wnl.0000244436.04036.a2 17130427

[4] Eriksson T Lidberg L Decreased plasma ratio of tryptophan to competing large neutral amino acids in human immunodeficiency virus type 1 infected subjects: possible implications for development of neuro-psychiatric disorders. J Neural Transm (1996) 103:157–64. 10.1007/BF01292624 9026369

[5] Longatti P Perin A Comai S Bertazzo A Rizzo V Costa CV A study of tryptophan metabolism *via* serotonin in ventricular cerebrospinal fluid in HIV-1 infection using a neuroendoscopic technique. Curr HIV Res (2007) 5:267–72. 10.2174/157016207780077020 17346140

[6] Hammoud DA Endres CJ Hammond E Uzuner O Brown A Nath A Imaging serotonergic transmission with [11C]DASB-PET in depressed and non-depressed patients infected with HIV. Neuroimage (2010) 49:2588–95. 10.1016/j.neuroimage.2009.10.037 PMC2818313 19853044

[7] Su S Zhao J Bremner JD Miller AH Tang W Bouzyk M Serotonin transporter gene, depressive symptoms, and interleukin-6. Circ Cardiovasc Genet (2009) 2:614–20. 10.1161/CIRCGENETICS.109.870386 PMC2802220 20031642

[8] Couch Y Anthony DC Dolgov O Revischin A Festoff B Santos AI Microglial activation, increased TNF and SERT expression in the prefrontal cortex define stress-altered behaviour in mice susceptible to anhedonia. Brain Behav Immun (2013) 29:136–46. 10.1016/j.bbi.2012.12.017 23305936

[9] Latorre E Mendoza C Matheus N Castro M Grasa L Mesonero JE IL-10 modulates serotonin transporter activity and molecular expression in intestinal epithelial cells. Cytokine (2013) 61:778–4. 10.1016/j.cyto.2013.01.012 23410504

[10] Malynn S Campos-Torres A Moynagh P Haase J The pro-inflammatory cytokine TNF-alpha regulates the activity and expression of the serotonin transporter (SERT) in astrocytes. Neurochem Res (2013) 38:694–704. 10.1007/s11064-012-0967-y 23338678

[11] Hsu JW Lirng JF Wang SJ Lin CL Yang KC Liao MH Association of thalamic serotonin transporter and interleukin-10 in bipolar I disorder: a SPECT study. Bipolar Disord (2014) 16:241–8. 10.1111/bdi.12164 24372850

[12] Chou YH Hsieh WC Chen LC Lirng JF Wang SJ Association between the serotonin transporter and cytokines: implications for the pathophysiology of bipolar disorder. J Affect Disord (2016) 191:29–35. 10.1016/j.jad.2015.10.056 26630394

[13] Wu F Kirmaier A Goeken R Ourmanov I Hall L Morgan JS TRIM5 alpha drives SIVsmm evolution in rhesus macaques. PLoS Pathog (2013) 9:e1003577. 10.1371/journal.ppat.1003577 23990789 PMC3749954

[14] Matsuda K Dang Q Brown CR Keele BF Wu F Ourmanov I Characterization of simian immunodeficiency virus (SIV) that induces SIV encephalitis in rhesus macaques with high frequency: role of TRIM5 and major histocompatibility complex genotypes and early entry to the brain. J Virol (2014) 88:13201–11. 10.1128/JVI.01996-14 PMC4249079 25187546

[15] Wu F Ourmanov I Riddick N Matsuda K Whitted S Plishka RJ TRIM5alpha restriction affects clinical outcome and disease progression in simian immunodeficiency virus-infected rhesus macaques. J Virol (2015) 89:2233–40. 10.1128/JVI.02978-14 PMC4338887 25473059

[16] Matsuda K Riddick NE Lee CA Puryear SB Wu F Whitted S A SIV molecular clone that targets the CNS and induces neuroAIDS in rhesus macaques. PLoS Pathog (2017) 13:e1006538. 10.1371/journal.ppat.1006538 28787449 PMC5560746

[17] Wienhard K Schmand M Casey ME Baker K Bao J Eriksson L The ECAT HRRT: performance and first clinical application of the new High Resolution Research Tomograph. Nucl Sci IEE ETrans (2002) 49:104–10. 10.1109/TNS.2002.998689

[18] Wilson AA Ginovart N Hussey D Meyer J Houle S In vitro and *in vivo* characterisation of [11C]-DASB: a probe for *in vivo* measurements of the serotonin transporter by positron emission tomography. Nucl Med Biol (2002) 29:509–15. 10.1016/S0969-8051(02)00316-5 12088720

[19] Meyer JH Wilson AA Ginovart N Houle S Misunderstandings about how to choose a reference region. Biol Psychiatry (2007) 61:1314. author reply 1314–1315. 10.1016/j.biopsych.2006.06.007 16989780

[20] Lammertsma AA Hume SP Simplified reference tissue model for PET receptor studies. Neuroimage (1996) 4:153–8. 10.1006/nimg.1996.0066 9345505

[21] Schmittgen TD Livak KJ Analyzing real-time PCR data by the comparative C(T) method. Nat Protoc (2008) 3:1101–8. 10.1038/nprot.2008.73 18546601

[22] Shah S Bohsali A Ahlbrand SE Srinivasan L Rathinam VA Vogel SN Cutting edge: *Mycobacterium tuberculosis* but not nonvirulent mycobacteria inhibits IFN-beta and AIM2 inflammasome–dependent IL-1beta production *via* its ESX-1 secretion system. J Immunol (2013) 191:3514–8. 10.4049/jimmunol.1301331 PMC3799997 23997220

[23] Jung M Kadam S Xiong W Rauch TA Jin SG Pfeifer GP MIRA-seq for DNA methylation analysis of CpG islands. Epigenomics (2015) 7:695–706. 10.2217/epi.15.33 25881900 PMC4607651

[24] Li X Baker-Andresen D Zhao Q Marshall V Bredy TW Methyl CpG binding domain ultra-sequencing: a novel method for identifying inter-individual and cell-type–specific variation in DNA methylation. Genes Brain Behav (2014) 13:721–31. 10.1111/gbb.12150 24954855

[25] Varnas K Halldin C Hall H Autoradiographic distribution of serotonin transporters and receptor subtypes in human brain. Hum Brain Mapp (2004) 22:246–60. 10.1002/hbm.20035 PMC6872082 15195291

[26] Meyer JH Imaging the serotonin transporter during major depressive disorder and antidepressant treatment. J Psychiatry Neurosci (2007) 32:86–102. 17353938 PMC1810585

[27] Ciesla JA Roberts JE Meta-analysis of the relationship between HIV infection and risk for depressive disorders. Am J Psychiatry (2001) 158:725–30. 10.1176/appi.ajp.158.5.725 11329393

[28] Arseniou S Arvaniti A Samakouri M HIV infection and depression. Psychiatry Clin Neurosci (2014) 68:96–109. 10.1111/pcn.12097 24552630

[29] Mills JC Pence BW Todd JV Bengtson AM Breger TL Edmonds A Cumulative burden of depression and all-cause mortality in women living with HIV. Clin Infect Dis (2018) 67(10):1575–81. 10.1093/cid/ciy264 PMC6206117 29618020

[30] Yoo-Jeong M Waldrop-Valverde D Mccoy K Ownby RL A structural equation model of HIV-related symptoms, depressive symptoms, and medication adherence. J HIV AIDS (2016) 2(3):1–15. 10.16966/2380-5536.123 PMC5042325 27695710

[31] Horberg MA Silverberg MJ Hurley LB Towner WJ Klein DB Bersoff-Matcha S Effects of depression and selective serotonin reuptake inhibitor use on adherence to highly active antiretroviral therapy and on clinical outcomes in HIV-infected patients. J Acquir Immune Defic Syndr (2008) 47:384–90. 10.1097/QAI.0b013e318160d53e 18091609

[32] Farinpour R Miller EN Satz P Selnes OA Cohen BA Becker JT Psychosocial risk factors of HIV morbidity and mortality: findings from the Multicenter AIDS Cohort Study (MACS). J Clin Exp Neuropsychol (2003) 25:654–70. 10.1076/jcen.25.5.654.14577 12815503

[33] Cannon DM Ichise M Rollis D Klaver JM Gandhi SK Charney DS Elevated serotonin transporter binding in major depressive disorder assessed using positron emission tomography and [11C]DASB; comparison with bipolar disorder. Biol Psychiatry (2007) 62:870–7. 10.1016/j.biopsych.2007.03.016 17678634

[34] Boileau I Warsh JJ Guttman M Saint-Cyr JA Mccluskey T Rusjan P Elevated serotonin transporter binding in depressed patients with Parkinson’s disease: a preliminary PET study with [11C]DASB. Mov Disord (2008) 23:1776–80. 10.1002/mds.22212 18661545

[35] Meyer JH Houle S Sagrati S Carella A Hussey DF Ginovart N Brain serotonin transporter binding potential measured with carbon 11–labeled DASB positron emission tomography: effects of major depressive episodes and severity of dysfunctional attitudes. Arch Gen Psychiatry (2004) 61:1271–9. 10.1001/archpsyc.61.12.1271 15583118

[36] Zhang LC Zeng YM Ting J Cao JP Wang MS The distributions and signaling directions of the cerebrospinal fluid contacting neurons in the parenchyma of a rat brain. Brain Res (2003) 989:1–8. 10.1016/S0006-8993(03)03123-8 14519505

[37] Lawson MA Kelley KW Dantzer R Intracerebroventricular administration of HIV-1 Tat induces brain cytokine and indoleamine 2,3-dioxygenase expression: a possible mechanism for AIDS comorbid depression. Brain Behav Immun (2011) 25:1569–75. 10.1016/j.bbi.2011.05.006 PMC3191256 21620953

[38] Fu X Lawson MA Kelley KW Dantzer R HIV-1 Tat activates indoleamine 2,3 dioxygenase in murine organotypic hippocampal slice cultures in a p38 mitogen-activated protein kinase–dependent manner. J Neuroinflammation (2011) 8:88. 10.1186/1742-2094-8-88 21810259 PMC3162509

[39] Abdel-Hameed EA Ji H Shata MT HIV-induced epigenetic alterations in host cells. Adv Exp Med Biol (2016) 879:27–38. 10.1007/978-3-319-24738-0_2 26659262

[40] Levine AJ Quach A Moore DJ Achim CL Soontornniyomkij V Masliah E Accelerated epigenetic aging in brain is associated with pre-mortem HIV-associated neurocognitive disorders. J Neurovirol (2016) 22:366–75. 10.1007/s13365-015-0406-3 PMC4900944 26689571

[41] Nelson KN Hui Q Rimland D Xu K Freiberg MS Justice AC Identification of HIV infection-related DNA methylation sites and advanced epigenetic aging in HIV-positive, treatment-naive U.S. Aids (2017) 31:571–5. 10.1097/QAD.0000000000001360 PMC5263111 27922854

[42] Nikolova YS Hariri AR Can we observe epigenetic effects on human brain function? Trends Cogn Sci (2015) 19:366–73. 10.1016/j.tics.2015.05.003 PMC4486509 26051383

[43] Kader F Ghai M Maharaj L The effects of DNA methylation on human psychology. Behav Brain Res (2017) 346:47–65. 10.1016/j.bbr.2017.12.004 29237550

[44] Schneider I Kugel H Redlich R Grotegerd D Burger C Burkner PC Association of serotonin transporter gene AluJb methylation with major depression, amygdala responsiveness, 5-HTTLPR/rs25531 polymorphism, and stress. Neuropsychopharmacology (2017) 43(6):1308–16. 10.1038/npp.2017.273 PMC5916353 29114103

[45] Uchida S Yamagata H Seki T Watanabe Y Epigenetic mechanisms of major depression: targeting neuronal plasticity. Psychiatry Clin Neurosci (2017) 72(4):212–27. 10.1111/pcn.12621 29154458

[46] Yip L Su L Sheng D Chang P Atkinson M Czesak M Deaf1 isoforms control the expression of genes encoding peripheral tissue antigens in the pancreatic lymph nodes during type 1 diabetes. Nat Immunol (2009) 10:1026–33. 10.1038/ni.1773 PMC2752139 19668219

[47] Barker HE Smyth GK Wettenhall J Ward TA Bath ML Lindeman GJ Deaf-1 regulates epithelial cell proliferation and side-branching in the mammary gland. BMC Dev Biol (2008) 8:94. 10.1186/1471-213X-8-94 18826651 PMC2570686

[48] Ordureau A Enesa K Nanda S Le Francois B Peggie M Prescott A DEAF1 is a Pellino1-interacting protein required for interferon production by Sendai virus and double-stranded RNA. J Biol Chem (2013) 288:24569–80. 10.1074/jbc.M113.479550 PMC3750155 23846693

[49] Barr SD Smiley JR Bushman FD The interferon response inhibits HIV particle production by induction of TRIM22. PLoS Pathog (2008) 4:e1000007. 10.1371/journal.ppat.1000007 18389079 PMC2279259

[50] Yan N Regalado-Magdos AD Stiggelbout B Lee-Kirsch MA Lieberman J The cytosolic exonuclease TREX1 inhibits the innate immune response to human immunodeficiency virus type 1. Nat Immunol (2010) 11:1005–13. 10.1038/ni.1941 PMC2958248 20871604

[51] Michelson RJ Collard MW Ziemba AJ Persinger J Bartholomew B Huggenvik JI Nuclear DEAF-1–related (NUDR) protein contains a novel DNA binding domain and represses transcription of the heterogeneous nuclear ribonucleoprotein A2/B1 promoter. J Biol Chem (1999) 274:30510–9. 10.1074/jbc.274.43.30510 10521432

[52] Lemonde S Turecki G Bakish D Du L Hrdina PD Bown CD Impaired repression at a 5-hydroxytryptamine 1A receptor gene polymorphism associated with major depression and suicide. J Neurosci (2003) 23:8788–99. 10.1523/JNEUROSCI.23-25-08788.2003 PMC6740417 14507979

[53] Szewczyk B Albert PR Burns AM Czesak M Overholser JC Jurjus GJ Gender-specific decrease in NUDR and 5-HT1A receptor proteins in the prefrontal cortex of subjects with major depressive disorder. Int J Neuropsychopharmacol (2009) 12:155–68. 10.1017/S1461145708009012 PMC2645471 18561871

[54] Czesak M Lemonde S Peterson EA Rogaeva A Albert PR Cell-specific repressor or enhancer activities of Deaf-1 at a serotonin 1A receptor gene polymorphism. J Neurosci (2006) 26:1864–71. 10.1523/JNEUROSCI.2643-05.2006 PMC6793620 16467535

[55] Drevets WC Thase ME Moses-Kolko EL Price J Frank E Kupfer DJ Serotonin-1A receptor imaging in recurrent depression: replication and literature review. Nucl Med Biol (2007) 34:865–77. 10.1016/j.nucmedbio.2007.06.008 PMC2702715 17921037

[56] Boldrini M Underwood MD Mann JJ Arango V Serotonin-1A autoreceptor binding in the dorsal raphe nucleus of depressed suicides. J Psychiatr Res (2008) 42:433–42. 10.1016/j.jpsychires.2007.05.004 PMC2268626 17574270

[57] Bose SK Mehta MA Selvaraj S Howes OD Hinz R Rabiner EA Presynaptic 5-HT1A is related to 5-HTT receptor density in the human brain. Neuropsychopharmacology (2011) 36:2258–65. 10.1038/npp.2011.113 PMC3176562 21750580

[58] Ase AR Reader TA Hen R Riad M Descarries L Regional changes in density of serotonin transporter in the brain of 5-HT1A and 5-HT1B knockout mice, and of serotonin innervation in the 5-HT1B knockout. J Neurochem (2001) 78:619–30. 10.1046/j.1471-4159.2001.00437.x 11483665

[59] Tylee DS Kawaguchi DM Glatt SJ On the outside, looking in: a review and evaluation of the comparability of blood and brain “-omes”. Am J Med Genet B Neuropsychiatr Genet (2013) 162b:595–603. 10.1002/ajmg.b.32150 24132893

