# Supplementary material for: Potential Mechanism for HIV-Associated Depression: Upregulation of Serotonin Transporters in SIV-Infected Macaques Detected by 11C-DASB PET
Source: Front Psychiatry. 2019 May 23;10:362. doi: 10.3389/fpsyt.2019.00362 (PMC6543249; doi:10.3389/fpsyt.2019.00362)
Supplement: Table 1S — Characteristics of group A animals. [file DataSheet_1.docx]

Table 1S: Characteristics of group A animals

| **Group A animals** | | | | | | | |
| --- | --- | --- | --- | --- | --- | --- | --- |
| **Animal ID** | **Imaging span (weeks)** | **Number of PET scans** | **Disease progression** | **Plasma VL (at last imaging session)**  **(SIV-RNA copies/ml)** | **CSF VL (at last imaging session)**  **(SIV-RNA copies/ml)** | **Inoculum** | **Genotype** |
| **SIV#1** | Baseline to 39 weeks p.i. | 4 | Rapid progressor | 6.10x10^5 | 2.50x10^3 | SIVsm804E | Q/Q |
| **SIV#2** | Baseline to 32 weeks p.i. | 4 | Rapid progressor | 1.24x10^5 | 1.63x10^4 | SIVsm804E | Q/Q |
| **SIV#3** | Baseline to 47 weeks p.i. | 5 | Rapid progressor | 4.95x10^5 | 1.81x10^6 | SIVsm804E | Q/Q |
| **SIV#4** | Baseline to 6 weeks p.i. | 2 | Rapid progressor | 4.08x10^7 | 2.18x10^7 | SIVsm804E | Q/Q |
| **SIV#5** | Baseline to 66 weeks p.i. | 5 | Slow progressor | 3.94x10^5 | 4.22x10^4 | SIVsm804E | Q/Q |
| **SIV#6** | Baseline to 93 weeks p.i. | 5 | Slow progressor | 4.35x10^5 | 4.02x10^7 | SIVsm804E | Q/Q |
| **SIV#7** | Baseline to 7 weeks p.i. | 2 | Rapid progressor | 2.73x10^5 | 2.6x10^3 | SIVsm804E | Q/Q |

Table 2S: Characteristics of group B animals

| **Group B animals** | | | | | | |
| --- | --- | --- | --- | --- | --- | --- |
| **Animal ID** | **Survival (weeks)** | **Plasma VL**  **(SIV-RNA copies/ml)** | **CSF VL (SIV-RNA copies/ml)** | **Clinical Symptoms and Neuropathology Results** | **Inoculum** | **Genotype** |
| **H813** | 79 | 5.16x10^5 | 1.22x10^5 | None | SIVsm804E | TFP/Q |
| **H881** | 49 | 1.0x10^6 | 1.2x10^4 | None | SIVsm804E-CL757 | TFP/Q |
| **H882** | 70 | 3.8x10^5 | 1.0x10^7 | None | SIVsm804E-CL757 | Q/Cyp |
| **H883** | 98 | 3.0x10^5 | 7.6x10^3 | None | SIVsm804E-CL757 | Q/Cyp |
| **H885** | 53 | 3.2x10^6 | 4.8x10^3 | None | SIVsm804E-CL757 | Q/Q |
| **H842** | 90 | 5.2x10^6 | 2.0x10^8 | None | SIVsm804E-CL757 | TFP/Q |
| **H819** | 119 | 1.98x10^6 | 4.1x10^2 | AIDS like disease without neurological signs | SIVsm804E | TFP/Q |
|  |  |  |  |  |  |  |
| **H814** | 15 | 2.4x10^6 | 2.74x10^7 | Neurological signs including tremors and motor difficulties. Positive neuropathology including widespread lesions characterized by perivascular cuffing and glial nodules containing multinucleated giant cells. | SIVsm804E | TFP/Q |
| **H816** | 16 | 9.08x10^5 | 2.07x10^6 | Neurological signs including tremors and motor difficulties. Positive neuropathology including widespread lesions characterized by perivascular cuffing and glial nodules containing multinucleated giant cells. | SIVsm804E | TFP/Q |
| **H817** | 47 | 6.33x10^4 | 9.6x10^3 | Positive neuropathology including glial nodules, perivascular cuffing with lymphocytes and macrophages and multinucleated giant cells. | SIVsm804E | TFP/Q |
| **H880** | 58 | 1.3x10^6 | 1.4x10^7 | Slow/staggered gait, arm tremors, hiding-hunched in back of the cage. Positive neuropathology including widespread lesions characterized by perivascular cuffing and glial nodules containing multinucleated giant cells. | SIVsm804E-CL757 | TFP/Q |
| **H886** | 54 | 3.2x10^6 | 4.6x10^6 | Ataxia, head pressing/holding, anisocoria, nystagmus. Positive neuropathology including widespread lesions characterized by perivascular cuffing and glial nodules containing multinucleated giant cells. | SIVsm804E-CL757 | Q/Q |
| **H887** | 54 | 2.4x10^6 | 1.6x10^8 | Ataxia, head pressing/holding, anisocoria, nystagmus, partial hind limb paralysis. Positive neuropathology including widespread lesions characterized by perivascular cuffing and glial nodules containing multinucleated giant cells. | SIVsm804E-CL757 | Q/Q |

Table 3S: Percent methylation of CpG islands in the SERT promoter region

|  | Sample | SERT-Methylation %- CpG island position | | | | | | | | | Average |
| --- | --- | --- | --- | --- | --- | --- | --- | --- | --- | --- | --- |
|  |  | 1 | 2 | 3 | 4 | 5 | 6 | 7 | 8 | 9 |  |
| SIV#1 | Baseline | 1 | 1 | 2 | 6 | 3 | 2 | 4 | 5 | 2 | **2.89** |
|  | 2 | 1 | 0 | 2 | 4 | 2 | 3 | 3 | 4 | 2 | **2.33** |
|  | Terminal | 1 | 1 | 2 | 4 | 2 | 2 | 2 | 3 | 2 | **2.11** |
| SIV # 2 | Baseline | 2 | 1 | 3 | 7 | 2 | 2 | 3 | 5 | 4 | **3.22** |
|  | 2 | 2 | 1 | 2 | 5 | 2 | 3 | 2 | 4 | 3 | **2.67** |
|  | 3 | 0 | 0 | 3 | 12 | 3 | 4 | 3 | 5 | 3 | **3.67** |
|  | Terminal | 1 | 2 | 3 | 6 | 3 | 4 | 2 | 4 | 2 | **3.00** |
| SIV # 3 | Baseline | 1 | 1 | 2 | 4 | 2 | 2 | 3 | 5 | 2 | **2.44** |
|  | 2 | 1 | 1 | 2 | 5 | 1 | 2 | 4 | 5 | 2 | **2.56** |
|  | 3 | 1 | 1 | 1 | 4 | 2 | 2 | 4 | 5 | 3 | **2.56** |
|  | Terminal | 1 | 1 | 2 | 3 | 2 | 2 | 2 | 5 | 2 | **2.22** |
| SIV # 4 | Baseline | 1 | 1 | 2 | 5 | 2 | 2 | 3 | 5 | 2 | **2.56** |
|  | 2 | 4 | 1 | 3 | 5 | 2 | 2 | 3 | 5 | 3 | **3.11** |
|  | Terminal | 1 | 1 | 1 | 4 | 1 | 1 | 2 | 3 | 2 | **1.78** |
| SIV # 5 | Baseline | 1 | 1 | 2 | 4 | 1 | 2 | 3 | 4 | 2 | **2.22** |
|  | 1 | 1 | 1 | 2 | 6 | 2 | 3 | 3 | 4 | 3 | **2.78** |
|  | 2 | 1 | 1 | 2 | 4 | 1 | 2 | 2 | 4 | 3 | **2.22** |
|  | Terminal | 1 | 1 | 2 | 5 | 2 | 3 | 3 | 6 | 2 | **2.78** |
| SIV # 6 | Baseline | 3 | 1 | 1 | 4 | 1 | 1 | 1 | 3 | 1 | **1.78** |
|  | 2 | 0 | 1 | 1 | 6 | 1 | 0 | 1 | 1 | 2 | **1.44** |
|  | 3 | 1 | 1 | 2 | 4 | 1 | 2 | 4 | 5 | 2 | **2.44** |
|  | Terminal | 1 | 1 | 2 | 4 | 1 | 2 | 3 | 4 | 1 | **2.11** |
| SIV # 7 | Baseline | 3 | 1 | 2 | 4 | 2 | 2 | 3 | 5 | 3 | **2.78** |
|  | 2 | 1 | 1 | 2 | 5 | 2 | 3 | 3 | 5 | 2 | **2.67** |
|  | Terminal | 1 | 1 | 3 | 6 | 1 | 4 | 5 | 5 | 2 | **3.11** |
